# Now, Later of Never: Multicenter Randomized Controlled Trial Call—Is Surgery Necessary after Atypical Breast Core Biopsy Results in Mammographic Screening Settings?

**DOI:** 10.1155/2015/192579

**Published:** 2015-04-21

**Authors:** Nikita Makretsov

**Affiliations:** The University of British Columbia, Vancouver, Canada V62 1Y6

## Abstract

Breast cancer mammographic screening leads to detection of premalignant and preinvasive lesions with an increasing frequency. Nevertheless, current epidemiologic evidence indicates that the screening reduces breast cancer specific mortality, but not overall mortality in breast cancer patients. The evidence is lacking whether aggressive eradication of DCIS (preinvasive form of breast carcinoma) by surgery and radiation is of survival benefit, as long-term breast cancer specific mortality in a cohort of patients with DCIS is already in a single digit percent range. Furthermore, it is currently not known whether the aggressive surgical eradication of atypical breast lesions which fall short of diagnosis of DCIS is of any benefit for the patients. Here we propose a model for a randomized controlled trial to generate high level evidence and solve this dilemma.

## 1. Introduction and Background

Mammographic breast cancer screening is under scrutiny as there is no firm evidence that it reduces mortality in female population. At the same time there is growing evidence that it leads to overtreatment due to false positive results [1, 2]. False positives include those patients with positive mammograms, who underwent breast core biopsy and were found to have no cancer by pathology. Meanwhile 3–5% of all breast core biopsies performed in mammographic setting remain indeterminate and are labeled by pathologists as “atypical” breast lesions [3]. These results lead to diagnostic surgical excision of breast tissue with placement of needle wire in the area of concern in the breast. These procedures are complex, expensive, and invasive and invariably cause stress and anxiety in patients. The efficiency of these procedures is measured in new cancers detected and is estimated to be 2–15% of all patients who undergo such procedures. Of these cancers, 85–90% are preinvasive ductal carcinoma in situ, associated with excellent outcomes. The remainder 5–10% cases are true invasive carcinomas, of which more than 80% have excellent prognostic features (3–5% risk of 5-year mortality) [4]. Thus, even the most conservative estimates show significant overdiagnosis and overtreatment as a result of this approach [1, 2]. Randomized controlled trials have never yet been proposed to explore the equivalence of conservative management of atypical breast lesions. It is time to explore conservative watchful management of atypical preinvasive breast lesions similar to preclinical prostate cancers, already adapted by medical community [5]. The aim of this short essay is to propose an overall design for this noninferiority trial.

## 2. Research Question, PICO (Participants, Intervention, Comparison, and Outcome)

Is watchful conservative management equivalent to surgical management atypical breast lesions in mammographic screening settings?

### 2.1. Participants

#### 2.1.1. Inclusion Criteria, Patients

Inclusion criteria are as follows:

- females in UK, US, and Canada, aged 50–70 (regular mammographic screening age group),
- any ethnicity,
- mammographic screening participant with mammography positive result, that is, nonpalpable abnormality or indeterminate calcifications by screening mammogram, requiring biopsy,
- pathology biopsy result indicating at least one of the following (in the absence of invasive carcinoma): atypical ductal hyperplasia, atypical lobular hyperplasia, flat epithelial atypia, breast papilloma with atypia, breast fibroadenoma with atypia, and ductal carcinoma in situ of low grade, and their synonyms, as outlined by Pinder [6],
- no prior diagnosis of breast cancer (either ipsi- or contralateral),
- no family history of breast cancer.

#### 2.1.2. Inclusion Criteria, Clinical Centers

The procedures should be performed at the participating centers with specialized breast unit with multidisciplinary breast clinical management team in place (including radiology, pathology, and surgery), which follow best practice guidelines and participate in appropriate quality assurance schemes [6–8].

#### 2.1.3. Exclusion Criteria, Patients

Exclusion criteria are as follows:

- clinically diagnosed or self-diagnosed palpable breast masses,
- any masses diagnosed by screening mammography,
- core biopsy diagnosis of invasive carcinoma of any type or high grade ductal carcinoma in situ, or diagnosis of encapsulated papillary carcinoma,
- patients with family history of breast cancer or genetic conditions associated with higher risk of breast cancer,
- patients with previously diagnosed and treated breast cancers,
- patients with other malignancies, except noninvasive skin cancers,
- patients who are unfit for surgery.

#### 2.1.4. Exclusion Criteria, Clinical Centers

The clinical centers which do not have organized breast units or multidisciplinary breast service in place or perform breast surgery occasionally are excluded.

### 2.2. Intervention

The main idea behind this trial design is evaluation of conservative management of atypical breast lesions detected by core biopsy by annual mammographic follow-up. Therefore, conservative management constitutes “intervention” in this trial. This should not be confused with standard care (i.e., surgical management), which is used for comparison.

### 2.3. Comparison

Current standard of care: needle wire radiologically localized surgical excision of the abnormal area in the breast, after core biopsy diagnosis of atypia.

### 2.4. Outcomes

*Primary Outcome*. Primary outcome is mortality (overall and breast cancer specific).

*Secondary Outcomes*. Secondary outcomes are quality of life score; follow-up mastectomy rates; economic measures such as cost per QALY [9]; rates and time to surgical excisional biopsy in intervention group.

## 3. Trial Design

*Design Type*. The type of the design is two arms, noninferiority randomized controlled trial.

*Allocation Concealment*. The recruiters will be shielded from allocation of the patients into study arms.

*Randomization*. It is a central internet based randomization with stratification by age.

*Blinding*. Challenges related to this trial are as follows: the patient blinding is not possible, as masking of surgery as a procedure is problematic. Leaving just a surgical scar without tissue removal appears nonethical and disfiguring for such sensitive body part as breast. Also, due to nature of nonpalpable breast lesions, they require placement of needle wire into the breast prior to surgery to guide surgical removal. Thus, the patient blinding is not at all possible. Surgeon blinding is also not possible for obvious reason. The radiologists will be blinded.

### 3.1. Sample Size

*Entry Assumption*. 10-year breast specific mortality in both groups could be estimated to be at least 2% (at least similar to DCIS of the breast).

If there is truly no difference between the standard and experimental management, then 1680 patients are required to be 80% sure that the limits of a two-sided 90% confidence interval will exclude a difference between the standard and experimental group of more than 2%. Online sample size calculator for equivalence trial with binary outcome measures was used (http://www.sealedenvelope.com/power/binary-equivalence/).

### 3.2. Feasibility

According to NHS data on breast screening [4] 1.7 million women were screened by mammography in England only in 2010. Of these, estimate of 7-8% had positive mammograms and required core biopsy (119.000–136.000 women). Of these, an estimate of 3–5% (3570–6800 women) has eligible atypical lesions after core biopsy pathology. Assuming that 50% of women will not consent to the study and prefer surgical excision, and additional 10% will not meet the eligibility criteria, the target study population could potentially be recruited into a multicenter trial within 1–3 years.

### 3.3. Follow-Up

There should be at least 10-year follow-up in order to reach conclusion, and this might appear problematic. Interim analysis can be performed at 5-year follow-up anniversary, if it is approved by the trial monitoring committee.

## 4. Trial Management

Multicenter trials have significant organizational challenges and require thorough planning and adequate funding. Planning committee should be established with representatives from all participating centers. Feasibility should be determined after thorough consideration of all potential risks and benefits. Coordinating center should be designated, responsible for randomization scheme delivery, trial management, data collection from all the centers, and data analysis. The organizational structure of the entire trial should be established, with all areas of responsibility and authority clearly declared. Steering and monitoring committees will need to be organized in such context and the standards of quality defined [10, 11].

## 5. Monitoring of Process and Other Specific Challenges

The patients in intervention and comparison arms will be followed up at the same mammographic intervals (once a year).

If the patient in the intervention arm is diagnosed with positive mammogram or by clinical self-examination during follow-up period, the needle wire localization surgical biopsy or lumpectomy will be performed, using similar methodology as in the comparison group. If diagnosis of invasive cancer or DCIS is established after such surgical excision, the women should undergo treatment as per current standard practice, similar to comparison group.

It is important that the radiologists and pathologists, who read the participants' follow-up investigations, remain blinded of prior mammograms and the initial core biopsy result for the entire period of trial.

## 6. Analysis

The data analysis should be performed by an intention to treat method.

*Primary Outcome*. The primary outcomes are breast cancer specific (Table 1) and overall mortality (not shown for brevity, due to similar approach). The 2 × 2 tables will have to be constructed and populated; 95% confidence intervals will be calculated as described earlier [12, 13]. In addition, Kaplan-Meier analysis with log-rank test could be performed, if the event rate will allow for a statistically valid comparison.

*Secondary Outcomes*. Quality of life score: generic 36-item short form quality of life instrument could be employed (Ware and Sherbourne, 1992, cited by [9]). The difference in scores between intervention and comparison group will be measured by* t*-test (if the normality assumptions will be met). Significance will be declared at *P* ≤ 0.05.

In addition, the mastectomy rate difference between intervention and comparison groups will be measured (Table 2).

## 7. Reporting

After completion, the trial results should be published in an open source medical venue.

## 8. Conclusion

If completed, this trial will provide high level evidence for justification for either conservative (watchful) management or surgical management of patients with atypical breast lesions.

## Figures and Tables

**Table 1:** Primary outcome, breast cancer specific mortality.

Death of breast cancer	Comparison (current surgical management)	Intervention arm (conservative management)
Event	*a*	*b*
No event	*c*	*d*
	Control event rate (CER) = *a*/(*a* + *c*)	Experimental event rate (EER) = *b*/(*b* + *d*)

Relative risk (RR): EER/CER (95% CI).

Absolute risk reduction (ARR, risk difference): EER − CER (95% CI).

Number needed to treat: 1/ARR.

**Table 2:** Secondary outcome: comparison of mastectomy rates.

Mastectomy	Comparison (current surgical management)	Intervention arm (conservative management)
Yes	*a*	*b*
No	*c*	*d*

The principles of statistical analysis of mastectomy rates difference are similar to the primary outcomes (i.e., relative risk, absolute risk reduction, and number needed to treat).
